# Low-Level Carotid Baroreceptor Stimulation Suppresses Ventricular Arrhythmias during Acute Ischemia

**DOI:** 10.1371/journal.pone.0109313

**Published:** 2014-10-06

**Authors:** Kai Liao, Lilei Yu, Kang Yang, Gaowa Saren, Songyun Wang, Bing Huang, Hong Jiang

**Affiliations:** Department of Cardiology, Renmin Hospital of Wuhan University, Wuhan, China; University of São Paulo, Brazil

## Abstract

**Background:**

The autonomic imbalance during acute ischemia is involved in the occurrence of life-threatening arrhythmias.

**Objective:**

To investigate the effect of autonomic nervous system (ANS) modulation by low-level carotid baroreceptor stimulation (LL-CBS) on ventricular ischemia arrhythmias.

**Methods:**

Anesthetized dogs were received either sham treatment (SHAM group, n = 10) or LL-CBS treatment (LL-CBS group, n = 10). The voltage lowering the blood pressure was used as the threshold for setting LL-CBS at 80% below the threshold. Treatment started 1 hour before left anterior descending coronary (LAD) occlusion, and continued until the end of experience. Ventricular effective refractory periods (ERP), monophasic action potential duration at 90% (APD_90_), ventricular arrhythmias, indices of heart rate variability, left stellate ganglion nerve activity (LSGNA) and infarct sizes were measured and analyzed.

**Results:**

Ventricular ischemia resulted in an acute reduction of blood pressure, which was not significantly affected by LL-CBS. After 1 hour of LL-CBS, there was a progressive and significant increase in ERP, increase in APD_90_, and decrease in LSGNA vs the SHAM group (all *P*<0.05). LL-CBS apparently reduced premature ventricular contractions (PVC, 264±165 in the SHAM group vs 60±37 in the LL-CBS group; *P*<0.01) during LAD occlusion. Number of episodes of ventricular fibrillation (VF) was 8 in the Control group versus 3 in the LL-CBS group (80% versus 30%, *P*<0.05). LL-CBS obviously increased high frequency (HF) component (*P*<0.05) and decreased low frequency/high frequency ratio (*P*<0.05) compared with the SHAM group. Ischemic size was not affected by LL-CBS between the two groups.

**Conclusions:**

LL-CBS reduced the occurrences of ventricular arrhythmias during acute ischemia without affecting blood pressure. The procedure was associated with changes of electrophysiological characteristics, nerve activity and heart rate variability. Therefore, LL-CBS may protect from ventricular arrhythmias during acute ischemic events by modulating ANS.

## Introduction

The autonomic imbalance, i.e. the activation of sympathetic nervous system and the reduction of parasympathetic activity, constitutes a fundamental element of acute myocardial ischemic pathophysiology [1]. It is associated with progressive ventricular remodeling, disease progression and arrhythmia generation. Particularly the autonomic imbalance increases the susceptibility of the heart to the fatal ventricular arrhythmias [2], [3], and modulation of autonomic tone using pharmacologic and nonpharmacologic means can reduce susceptibility to sudden cardiac death [4].

The carotid baroreflex circuit plays a critical role in blood pressure (BP) regulation via modulation of the sympathetic tone. Carotid baroreceptor stimulation (CBS) can reset autonomic tone, and were used as a promising therapeutic approach to treat refractory hypertension and heart failure [5], [6]. The potential mechanism of CBS may not just by sympathetic withdrawal, but also by increased vagal activation. It results in reduces whole sympathetic drive [7]. Sympathetic inhibition and/or parasympathetic activation seem to be protective for ventricular arrhythmias. Previous studies demonstrated the therapeutic effect of CBS on hypertension and heart failure [5], [6]. Few studies of CBS on arrhythmia has been reported. Recently we confirmed that CBS may have a beneficial impact on ventricular arrhythmias induced by acute ischemia through modulation of autonomic tone in dogs [8]. However, strong baroreflex stimulation usually lowers BP. In order to reduce dramatic fluctuations in BP, low-level carotid baroreceptor stimulation (LL-CBS) may be a good choice. As LL-CBS is at voltage substantially below that which lowed BP. Linz D et al [9] find LL-CBS result in moderate shortening in atrial refractoriness, and do not result in heart rate (HR) and BP reduction in normotensive pigs. The effects of LL-CBS on ventricular electrophysiological characteristics and ventricular arrhythmia remain unclear. We hypothesized that LL-CBS may reduce the arrhythmias induced by acute ischemia through modulating autonomic nervous system. The present study aimed to explore the effects of LL-CBS on ventricular electrophysiological characteristics in normal dog heart and the occurrences of ventricular arrhythmias during left anterior descending coronary (LAD) occlusion.

## Methods

### Animal preparation

The study protocol was approved by the Ethical Committee of the Wuhan University School of Medicine, and all animal handling was performed in accordance with the Wuhan Directive for Animal Research and the current Guidelines for the Care and Use of Laboratory Animals published by the National Institutes of Health (NIH publication no. 85–23, revised 1996). All surgery was performed under sodium pentobarbital anesthesia, and every effort was made to minimize suffering. 20 male mongrel dogs weighing 18±5 kg were supplied by the center of experimental animal in medical college of Wuhan University, and all owners of the dogs agreed to have their animals involved and provided a statement of informed consent. All dogs were randomized to either LL-CBS treatment (LL-CBS group, n = 10) or sham treatment including LL-CBS electrode implanting without stimulation (SHAM group, n = 10).

All dogs were anesthetized with sodium pentobarbital (30 mg/kg), and general anesthesia was maintained by hourly intravenous injection of 50–100 mg. Dogs were intubated and attached to positive pressure ventilation (MAO01746, Harvard Apparatus Holliston, USA) with a mixture of room air and 100% oxygen. Normal saline at 50 to 100 ml·h^−1^ was infused to replace spontaneous fluid losses from a femoral vein sheath. Arterial blood pressure was continuously monitored from a femoral arterial sheath. Body surface electrocardiogram (ECG) was recorded by using subcutaneous needle electrodes during the whole procedure using a computer-based Lab System (Lead 2000B, Jingjiang Inc., China). A heating pad was used to maintain the core body temperature of the dogs at 37.0±0.5°C.

### Carotid baroreceptor stimulation

**Figure 1:**
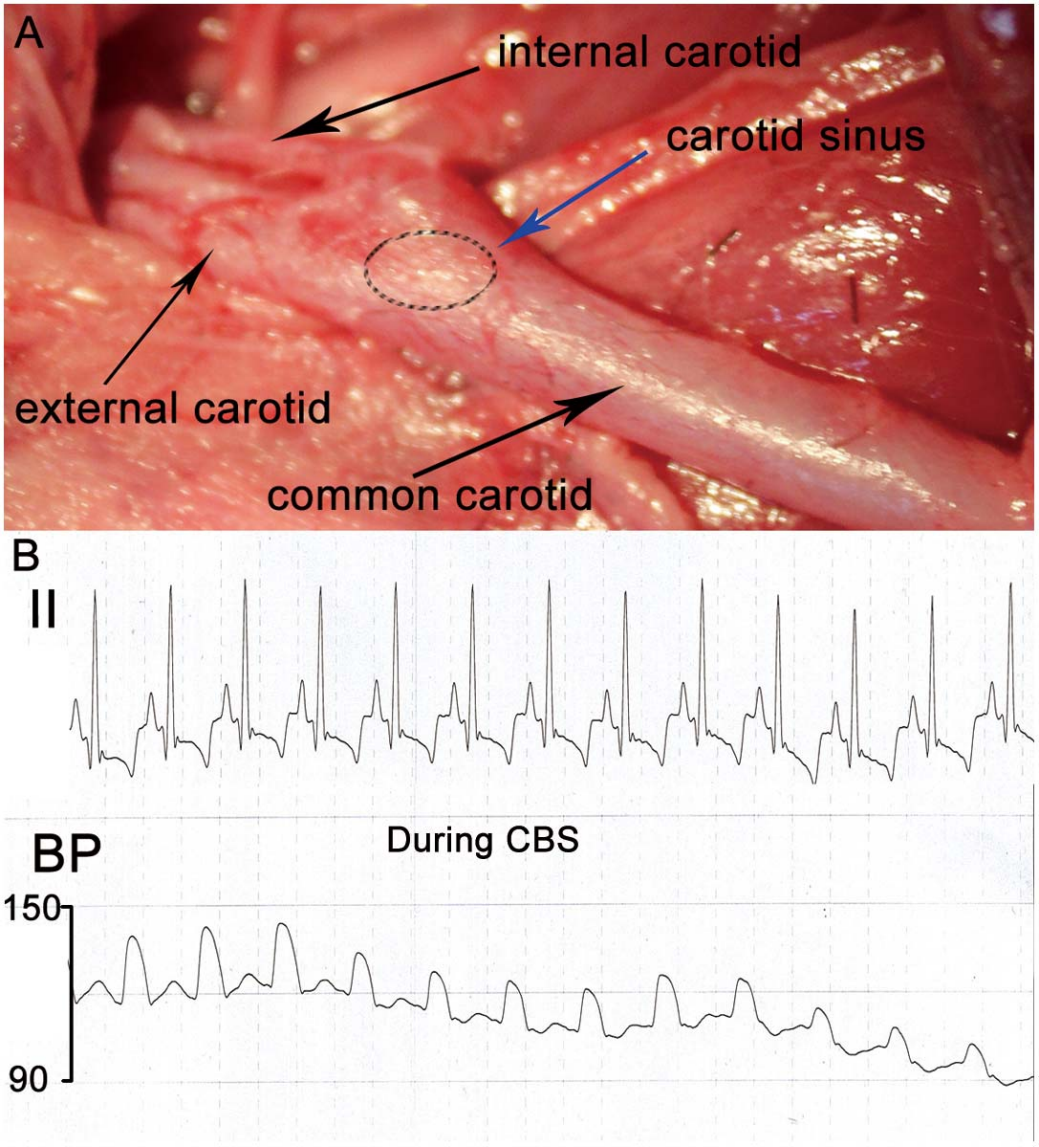
Representative examples of the location of the carotid sinus (A). All arteries were marked by the black arrow. The left carotid sinus was highlighted by the blue arrow. Representative changes of blood pressure and heart rate during CBS (B). It suggested that the electrode was implanted the proper placement of carotid sinus. CBS, carotid baroreceptor stimulation.

The right common carotid artery and internal carotid artery bifurcation was exposed and isolated in all dogs (Fig. 1A). A custom-made Ag-AgCl stimulating electrode was implanted circumferentially around carotid sinus and connected to the pulse generator (SEN-7103, Nihon Kohden, Tokyo, Japan). Proper placement of the electrode was confirmed by 3 to 4 acute stimulation runs performed 3 to 5 minutes apart and each confirming an acute drop of BP and a reduction of heart rate (HR) (Fig. 1B). After a break of about 15 minutes, BP and HR can usually be restored to pre-stimulus level. CBS was started again, and the voltage from 1 V to start. The voltage was increased 0.2 V per 5 minutes (50 Hz, 0.5-ms square wave) until reduction of the BP was achieved. The voltage necessary to achieve a reduction of the BP was used as the threshold for setting the LL-CBS in each experiment. The voltage was used as stimulation setting LL-CBS at 80% below the threshold. The stimulation threshold was checked every twenty minutes to ensure that LL-CBS was set appropriately. The pulse generator was programmed in LL-CBS group using the following parameters: 2.4–4.8 V, 50 Hz, and 0.5 ms pulse duration [5]. The stimulation was not stopped until the experiment was over in LL-CBS group. The stimulating electrode was implanted in proper placement without stimulation in SHAM group.

### Electrophysiological measurements

**Figure 2:**
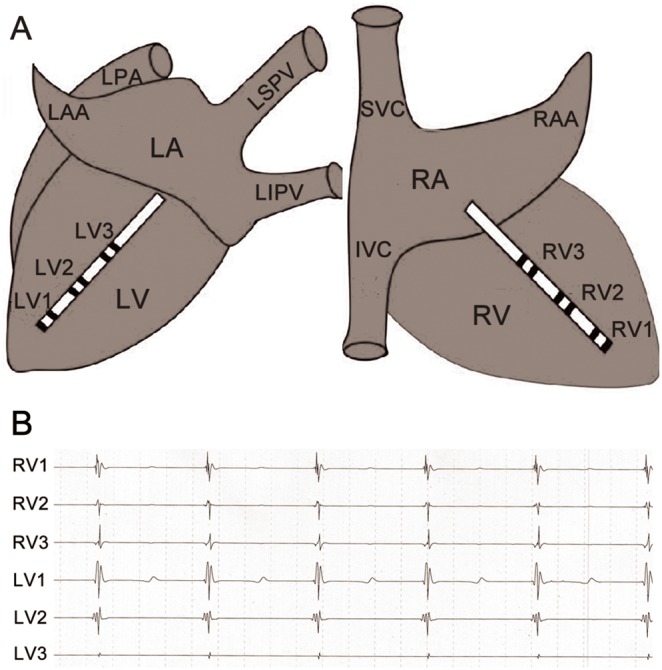
Schematic representation and catheter positions in the left and right (A) ventricular free walls and simultaneous epicardial electrocardiogram and BP recording (B). LV, left ventricle; RV, right ventricle; LA, left atrium; RA, right atrium; LAA, left atrial appendage; RAA, right atrial appendage; LSPV, left superior pulmonary vein; LIPV, left inferior pulmonary vein; PAT, pulmonary artery trunk; SVC, superior vena cava; IVC, inferior vena cava; LV1, LV2, LV3, RV1, RV2 and RV3 are three different sites from the apex (LV1 and RV1) to the base (LV3 and RV3) of the left and right ventricular free walls.

Both left and right thoracotomies were performed at the 5th intercostal space to expose the heart. Two multi-electrode catheters with 2 mm interelectrode distance were sutured to evaluate effective refractory periods (ERP) at 6 epicardial sites from the apex to the base in the left and right ventricular free walls, as described previously (Fig. 2) [10].

A custom-made Ag-AgCl electrode was sewn on the right atrial appendage for pacing atrium. Ventricular monophasic action potentials (MAP) were recorded from the endocardium of the right ventricle by a MAP catheter (Foehr Medical Instruments GMBH, Seeheim, Germany) during regular atrial pacing (BCL = 340 milliseconds). The monophasic action potentials were filtered at 0.05 to 1200 Hz and local cardiac electrograms between 30 and 500 Hz. The APD_90_ was defined as MAP measured at 90% repolarization. Changes in refractoriness and repolarization contribute greatly to proarrhythmic substrate. The changes of ERP and APD_90_ were measured during baseline and 1 hour in all animals.

### Acute ischemia protocol

1 hour after LL-CBS or sham treatment, the left anterior descending coronary arterys (LAD) were isolated in all dogs. LAD was occluded by ligature (3-0 silk) for 1 hour. Ligature was positioned at approximately half of the distance from the apex.

### Measurement of ventricular arrhythmias incidences

Ventricular arrhythmias were analyzed during 1 hour after LAD occlusion. PVC, ventricular tachycardia (VT), and VF were defined according to the Lambeth convention criteria with more rigorous modifications [7]. Specifically, PVC was defined as ventricular contractions without atrial depolarization. VT was defined as more than six consecutive PVC. VF was characterized by a loss of synchronicity of the electrocardiogram plus decreased amplitude and a precipitous fall in BP for >1 s.

### Heart rate variability (HRV) power spectral analysis

Spectral power for HRV was analyzed on 5-minute segments during baseline, 1 hour after treatment and 10 minute after LAD occlusion. An autoregressive algorithm was used to analyze digitized signals from the electrocardiographic recordings. The following power spectral variables were determined: high frequency component (HF, from 0.15 to 0.40 Hz); low frequency component (LF, from 0.04 to 0.15 Hz); and a very-low-frequency component (VLF, below 0.04 Hz). We calculated the ratio between LF and HF powers of RR variability (LF/HF). HF component is an index of parasympathetic activity, and LF/HF ratio is an index of sympathetic activity in HRV analysis [11]. HRV analysis was measured by a data acquisition system (LabChart Pro, ADInstruments Ltd, Sydney, NSW, Australia).

### Left stellate ganglion nerve activity (LSGNA) recordings

A pair of silver electrodes was inserted into the fat pad located at the left stellate ganglion (LSG). The electrodes would not be displaced by either cardiac or respiratory movement. The electrodes were connected by a common lead to a preamplifier (DP-304, Warner Instruments, Hamden, CT, USA). High-pass (50 Hz) filtering was used to reduce the electrocardiographic signals from the LSG recording channel. LSGNA was integrated at a time constant of 100 ms. The raw LSGNA was recorded with a PowerLab data acquisition system (LabChart Pro, ADInstruments Ltd, Sydney, NSW, Australia). The LSGNA was characterized by the recorded amplitude and frequency. Neural activity was defined as deflections with a signal-to-noise ratio greater than 3∶1 and the amplitude and frequency were manually determined as previously described [12], [13]. LSGNA is representative of cardiac sympathetic activity.

### Ischemic size determination

To delineate the ischemic from the nonischemic tissue, a dye exclusion method using Evans blue was applied at the end of the experiment. The left auricular appendage was cannulated. Through this cannula, 200 mL of an Evans blue solution (2% in physiological saline) was infused into the left atrium, left ventricle, aorta and coronary artery, which resulted in a dark blue staining of the nonischemic area. The heart was rapidly excised, removed the atria and right ventricular free wall. Then the heart was cut transversely into 0.5-cm thick slices. Both the nonstained ischemic areas and the blue-stained normal areas of the left ventricular free wall and of the septum were cut out and weighed separately. The mass of the ischemic tissue was expressed as fraction of the left, or septal ventricular tissue mass [14].

### Statistical analysis

All continuous variables were expressed as mean ± standard deviation. Two way ANOVA was used to compare the mean of ERP, APD_90_, HF, LF/HF, LSGNA between the SHAM group and the LL-CBS group. The independent-samples t-test was used to compare the mean of PVC, VT and infarct size between two groups. Fisher’s exact test was used to compare the incidence of VF. GarphPad Prism 5.01 for Windows (GarphPad Software Inc., La Jolla, CA, USA) was used for statistical analysis. A value of *P*<0.05 was considered significant for the differences tested.

## Results

### Changes of hemodynamics

The values of systolic blood pressure (SBP), diastolic blood pressure (DBP) and HR were not significantly different between the two groups during baseline, 1 hour and LAD occlusion (Table 1, all *P*>0.05). For example, SBP were no significant difference between the two groups during baseline (133±11 mmHg in the SHAM group vs 127±14 mmHg in the LL-CBS group, P>0.05). LL-CBS did not involve in the modulation of the SBP, DBP, and HR.

**Table 1:** Changes of hemodynamics.

	Baseline			1 hour			LAD occlusion		
	SHAM	LL-CBS	*P*	SHAM	LL-CBS	*P*	SHAM	LL-CBS	*P*
SBP (mmHg)	133±11	127±14	>0.05	132±11	123±15	>0.05	118±15	107±16	>0.05
DBP (mmHg)	104±12	94±11	>0.05	103±12	93±7	>0.05	87±9	76±10	>0.05
HR (beat/min)	144±17	135±12	>0.05	141±16	140±17	>0.05	157±13	150±12	>0.05

Table 1 Results were taken immediately at baseline, 1 hour and LAD occlusion. The values of SBP, DBP and HR were all not significantly different between the two groups. DBP, diastolic blood pressure; HR, heart rate; SBP, systolic blood pressure.

### Changes of ventricular ERP

**Figure 3:**
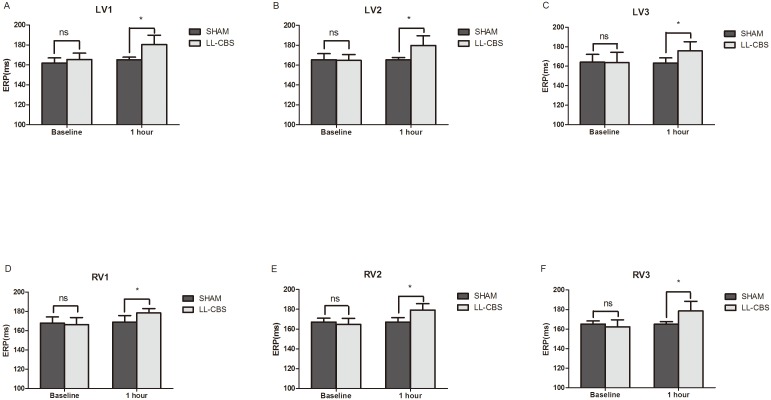
Ventricualr ERP at baseline and after 1 hour in the both two groups. Three different sites from the apex (LV1 and RV1) to the base (LV3 and RV3) of the ventricular free walls were recorded. LL-CBS did significantly prolong ERP compared with the SHAM group. ns, no significant; *P<0.01 when compared with the SHAM group.

Figure 3 summarizes ventricular ERP at 6 epicardial sites in all dogs during baseline and 1 hour. The baseline values of ERP were not significantly different among the two groups (*P*>0.05). However, LL-CBS did apparently prolong ERP after 1 hour treatment compared with the SHAM group (all *P*<0.01). For example at LV1 site, ERP were no significant difference between the two groups during baseline (162±5 mmHg in the SHAM group vs 165±6 mmHg in the LL-CBS group, P>0.05, Fig. 3A), and the values of the LL-CBS group were apparently higher than the SHAM group after 1 hour treatment (180±9 mmHg in the LL-CBS group vs 165±3 mmHg in the SHAM group, P<0.01, Fig. 3A).

### Changes of ventricular APD_90_

**Figure 4:**
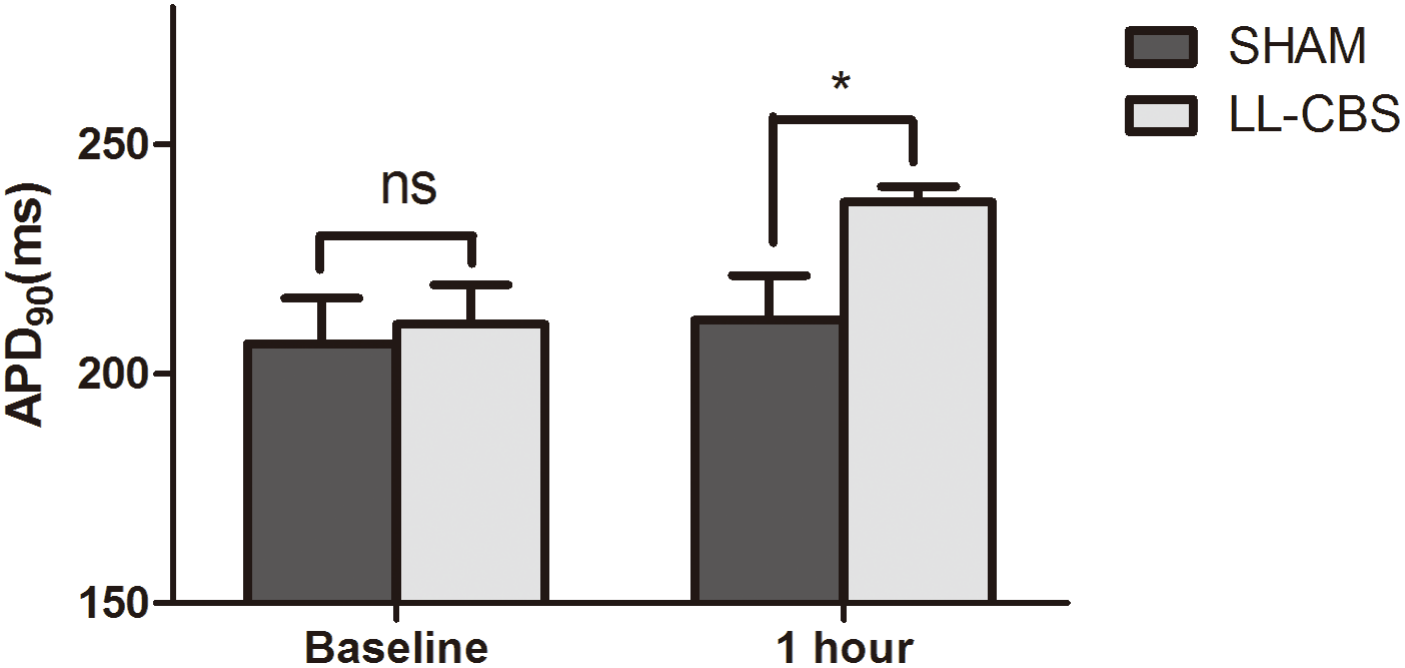
APD_90_ at baseline and after 1 hour in the both two groups. LL-CBS did significantly prolong APD_90_ compared with the SHAM group. ns, no significant; *, *P*<0.01 when compared with the SHAM group.

Figure 4 shows APD_90_ before and after 1 hour treatment in the both two groups. APD_90_ was not affected by the sham treatment (*P*>0.05). LL-CBS did significantly prolong APD_90_ compared with the SHAM group (238±3 ms in the LL-CBS group vs 212±10 mmHg in the SHAM group, P<0.01, Fig. 4).

### Ventricular arrhythmias

**Figure 5:**
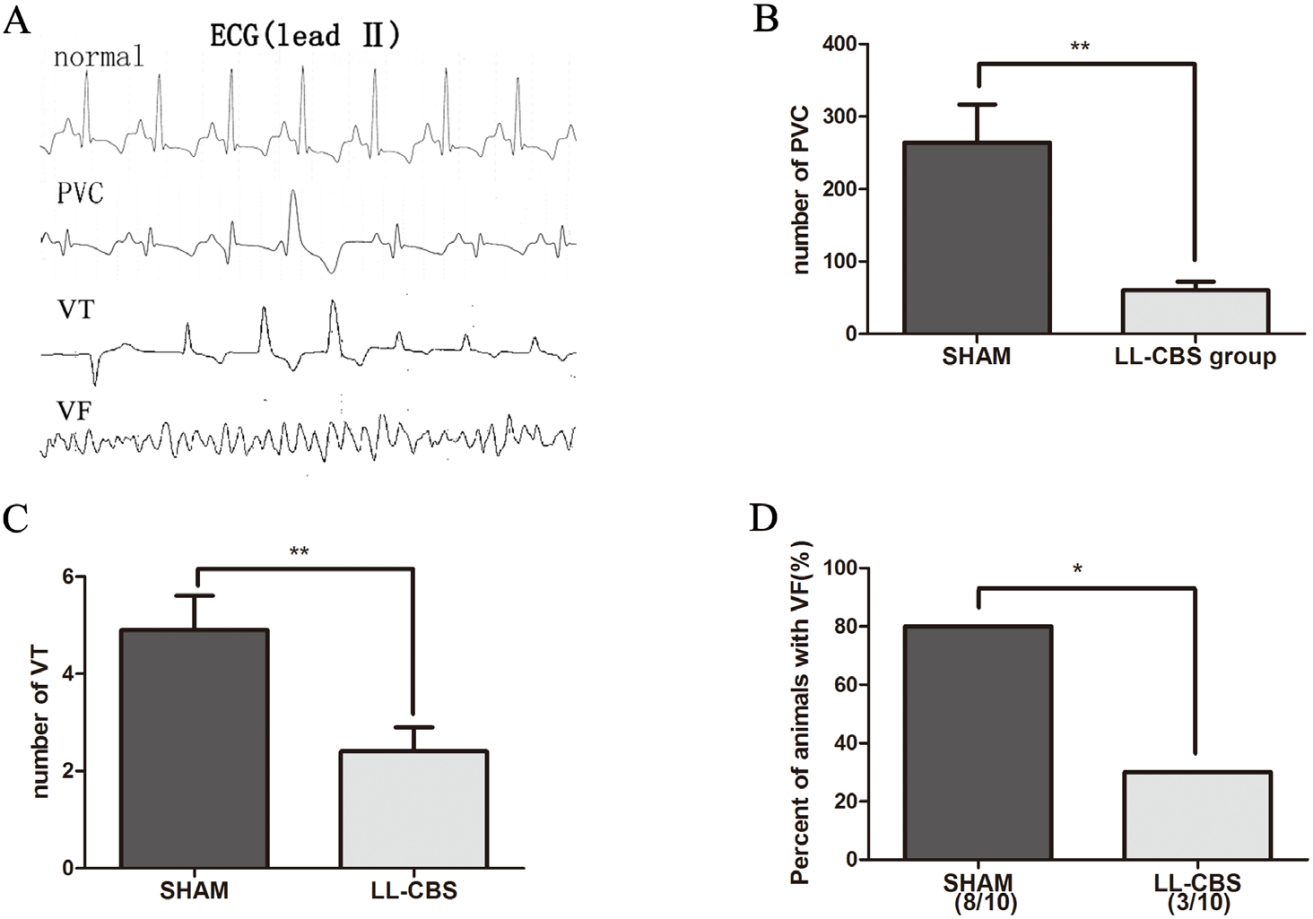
LL-CBS and the occurrence of cardiac arrhythmia after LAD occlusion. A shows examples of normal, PVC, VT and VF ECG (lead II) tracings. PVCs were markedly decreased in LL-CBS groups after LAD occlusion, compared with SHAM group (B). The number of VT episodes was significantly reduced in LL-CBS group after LAD occlusion, compared with SHAM group (C). Percentage of animals with VF was apparently reduced in BRS group after LAD occlusion, compared with SHAM group (D). Data are presented as mean±SD. PVC = premature ventricular contraction; VT = Ventricular tachycardia; VF = Ventricular fibrillation.

Figure 5A shows examples of normal, PVC, VT and VF ECG tracings. As shown in Figure 5B, 1 hour continuous electrocardiogram recording after LAD occlusion showed that the number of PVC in the LL-CBS group was significantly decreased compared with the SHAM group (60±37 in the LL-CBS group vs 264±165 in the SHAM group, *P*<0.01). Moreover, the number of VT episodes was significantly decreased in the LL-CBS group compared with the SHAM group (Fig. 5C) (2.4±1.6 in the LL-CBS group vs 4.9±2.2 in the SHAM group, *P*<0.01). VF occurred in 80% (8/10) of all shame-treated animals during LAD occlusion. By contrast, only 30% (3/10) of all LL-CBS-treated animals experienced VF (Fig. 5D) (*P*<0.05).

### Changes of HF and LF/HF

**Figure 6:**
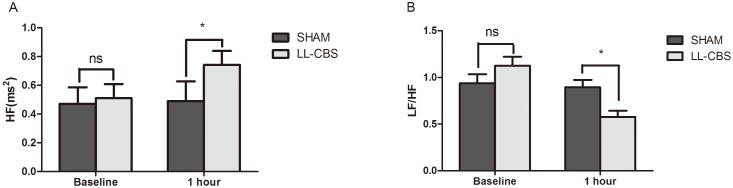
HF and LF/HF during baseline, 1 hour in the both two groups. Both HF (Fig. 6A) and LF/HF (Fig. 6B) were not significantly different between the two groups during the baseline status. LL-CBS obviously increased HF component and decreased LF/HF compared with the SHAM group. ns, no significant; *, *P*<0.01 when compared with the SHAM group.

Figure 6 shows changes of HF and LF/HF after 1 hour in the two groups. Both HF (Fig. 6A) and LF/HF (Fig. 6B) were not significantly different during the baseline status in the two groups. Conversely, HF value of the LL-CBS group was obviously higher than the SHAM group after 1 hour treatment (0.74±0.10 ms^2^ in the LL-CBS group vs 0.49±0.14 ms^2^ in the SHAM group, *P*<0.01). And LF/HF value of the LL-CBS group was apparently lower than the SHAM group after 1 hour treatment (0.58±0.21 in the LL-CBS group vs 0.89±0.26 in the SHAM group, *P*<0.01).

### Changes of LSGNA

**Figure 7:**
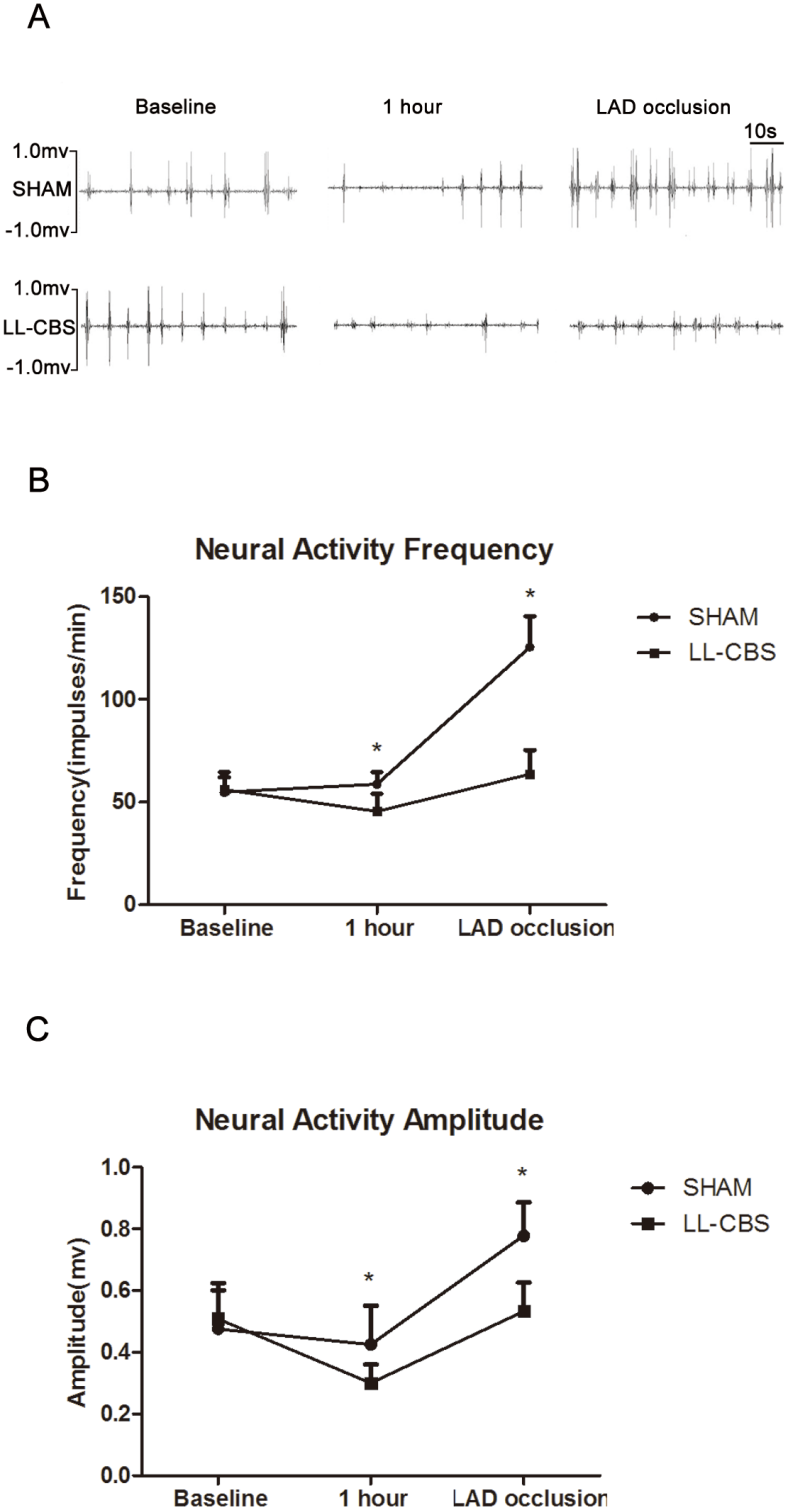
Changes of left stellate ganglion nerve activity. A: A typical example of neural recordings from the left stellate gangliona at baseline, 1 hour and LAD occlusion. B, C: The average amplitude and frequency of neural recordings in the both groups. LAD occlusion causes a progressive increase in the frequency and amplitude of neural firing in the two group, but, which partly increased in the LL-CBS group compared with the SHAM group. LL-CBS suppressed left stellate ganglion nerve activity. *P*<0.05 when compared with the SHAM group.

A typical example of the continuous monitoring of the neural activity recorded from the LSG at baseline, 1 hour and LAD occlusion is shown in Figure 7A. Figure 7B and 7C show LL-CBS causes a progressive decrease in the frequency (*P*<0.05) and amplitude (*P*<0.05) of neural firing compared to the SHAM group at 1 hour. LAD occlusion causes a progressive increase in the frequency and amplitude of neural firing in both group, but, which partly increased in the LL-CBS group compared with the SHAM group (*P*<0.01). LL-CBS suppressed LSGNA.

### Size of the ischemic area after LAD occlusion

A well-defined borderline was present between the ischemic zone and the nonischemic cardiac tissue. Table 2 summarized the left ventricle and septum myocardial mass of the ischemic area in the different groups. There was no significant difference between the two groups (*P*>0.05).

**Table 2:** Percentage of ischemic myocardial mass (%).

	Left ventricle	Septum
SHAM group (n = 10)	35.6±7.1	30.1±6.0
LL-CBS group (n = 10)	31.4±4.3	27.3±4.0
P	>0.05	>0.05

Table 2 summarized the left ventricle and septum myocardial mass of the ischemic area in the different groups. There was no significant difference between the two groups.

## Discussion

In this canine, randomized, acute myocardial ischemia model, the main findings were that LL-CBS suppressed PVC, VT and VF occurrences following LAD occlusion. Furthermore, this effect was associated with a prolongation of ventricular ERP and ventricular APD_90_, increased HF, decreased LF/HF and LSGNA.

Ventricular arrhythmias occurring early after induction of myocardial ischemia has been characterized in various animal models [15]. One hour after ischemia is a high incidence of arrhythmias time. In the present study, LL-CBS can reduce ventricular arrhythmias in this hour. Ventricular arrhythmias are important causes for sudden cardiac death, which is associated with autonomic imbalance [16] and reentry mechanism [17].

Heart was mainly regulated by ANS. The parasympathetic components originate in the vagus nerve. The sympathetic components originate primarily in the cervical spinal cord and also in the vagus nerve, which contains both sympathetic and parasympathetic fibers [18]. Recent study records sympathetic nerve activity and vagal nerve activity directly in myocardial ischemia model. The study shows myocardial ischemia results in increased autonomic imbalance [19]. In the present study HRV was used to reflect the body’s overall ANS activity. HF component is an index of parasympathetic activity, and LF/HF ratio is an index of sympathetic activity in HRV analysis [11]. The sympathetic tone increased and the vagal tone reduced according to HRV analysis during acute ischemia. In additional, the stellate ganglion is a sympathetic ganglion formed by the fusion of the inferior cervical ganglion and the first thoracic ganglion. LSG is a major source of cardiac sympathetic innervation. Lan S. Chen et al find the direct evidence supports a temporal relationship between spontaneously increased sympathetic discharges and cardiac arrhythmia in ambulatory animals [13]. That is the majority of malignant ventricular arrhythmias are preceded by LSG nerve activity in myocardial ischemia model dogs. Acute ischemia is usually accompanied by high sympathetic hyperactivity. While baroreceptors located in the heart and great vessels modulate the sympathetic tone and the vagal tone simultaneously [20]. We found LL-CBS increased HF, decreased LF/HF and LSGNA. CBS can not only make sympathetic withdrawal, but also increase vagal activity, then reduce whole sympathetic drive finally in a subgroup of patients with refractory arterial hypertension [6]. Our results mean LL-CBS reduced ischemia-induced ventricular arrhythmias in dog, accompanied by making sympathetic withdrawal and vagal activation. So modulation of ANS might be the antiarrhythmia mechanism of LL-CBS following.

Sympathetic activation following myocardial infarction contributes to adverse electrophysiologic remodeling, which results in heterogeneous ventricular repolarization and in a myocardial substrate conducive to lethal re-entrant arrhythmias [21]. ERP and APD_90_ elongation have been demonstrated to suppress re-entrant ventricular arrhythmias [22]. These changes are associated with relationship of sympathetic tone and vagal tone [3], [23]. In this study, LL-CBS can prolong ventricular ERP and APD_90_, and accompanied by reducing occurrences of VT and VF during LAD occlusion. So LL-CBS might also reduce re-entrant arrhythmias through modulating ANS in our study.

Adjustment of sympathetic tone and vagal tone has already been one target of suppressing ischemia induced ventricular arrhythmias. The effects of direct vagus nerve stimulation on myocardial ischemia induced arrhythmias have been confirmed in many studies [24], [25], [26]. Left or bilateral stellate ganglion blockade [13], [19], Renal denervation [27], spinal cord stimulation [28] and high thoracic epidural anesthesia [29] all show some protective effects against ischemia-induced arrhythmias. But these methods were not widely used in clinical practice. Difficulty operating, security, big damage and intolerable complications and side effects may be the main reasons. So development of a new strategy is urgently which is available to modulate the complex interaction between the autonomic nervous system and the heart, shows potential antiarrhythmic effect, and is easy to apply in clinical. The effect of LL-CBS on ischemia induced arrhythmias has been brought to our attention. As a way of physiological regulation, which will lead to a relatively few side effects and high safety. The most important thing is that CBS has already been applied in Rheos pivotal trial for the long-term treatment of resistant hypertension. Sustained efficacy, CBS safety, device safety and no significant effect on renal function have been testified [30], [31]. Yet we defined the voltage lowering the BP as threshold. Setting LL-CBS at 80% below the threshold had no significant effect on changes of BP and HR. LL-CBS might be a promising approach on acute myocardial infarction induced arrhythmias without through changes of HR and BP.

LL-CBS was not significant effect on infarct size during LAD occlusion. We found that there was not significant difference in the left ventricle and septum myocardial mass of the ischemic area among both groups. Motonori Ando also shows vagal stimulation can not induce significant changes myocardial infarcts in rat model of acute ischemia [24]. Jacob Odenstedt et al find spinal cord stimulation has no effect on infarct size or infarct size/area at risk in a porcine ischemia-reperfusion model, and presume more severe and prolonged ischemia are chief reasons [28]. But low-amplitude, left vagus nerve stimulation significantly attenuates infarct size through prevention of mitochondrial dysfunction during acute ischemia-reperfusion injury in swine [26]. Disparity of stimulation mode, sustain time and model species may be the major reasons. Further molecular mechanisms and signaling pathway studies may explain these differences.

### Limitations

We investigated acute effects of LL-CBS on ventricle electrophysiology in normal canine heart and acute ischemic arrhythmia. Acute electrophysiological effects of LL-CBS are relevant in the clinically setting, particularly at the beginning of treatment. But chronic LL-CBS might shows additional beneficial anti remodeling effects, which were not investigated in this study. To reduce experimental error, all dogs are used on the right carotid sinus stimulation. In ongoing studies left or bilateral LL-CBS should be evaluated. To limit the number of animals needed for the study, we performed electrophysiologic measurements and acute ischemia in the same dog. The invasive measurements may interference ventricular arrhythmias.

### Conclusion and perspectives

LL-CBS reduced the occurrences of ventricular arrhythmias during acute ischemia without affecting BP. The procedure was associated with changes of electrophysiological characteristics, HRV and neural activity. Therefore, LL-CBS may protect from ventricular arrhythmias during acute ischemic events by modulating ANS. In view of the present results, we can provide an alternative therapeutic strategy with a neural interface approach to ischemia-induced arrhythmias. LL-CBS regulates autonomic system by physiologic manner with no pacing, no ablation, no nerve damage. The therapy has been already applied in treatment of resistant hypertension and heart failure, so maybe will greatly beneficial for acute myocardial ischemia patients with hypertension and/or heart failure. Thus, ANS modulation by LL-CBS may be a safe option to protect patients at high risk for myocardial infarction from life-threatening arrhythmias.
